# Identification and Molecular Analysis of Four New Alleles at the *W1* Locus Associated with Flower Color in Soybean

**DOI:** 10.1371/journal.pone.0159865

**Published:** 2016-07-21

**Authors:** Jagadeesh Sundaramoorthy, Gyu Tae Park, Jeong Ho Chang, Jeong-Dong Lee, Jeong Hoe Kim, Hak Soo Seo, Gyuhwa Chung, Jong Tae Song

**Affiliations:** 1 School of Applied Biosciences, Kyungpook National University, Daegu, Republic of Korea; 2 Department of Biology Education, Kyungpook National University, Daegu, Republic of Korea; 3 Department of Biology, Kyungpook National University, Daegu, Republic of Korea; 4 Department of Plant Bioscience, Seoul National University, Seoul, Republic of Korea; 5 Department of Biotechnology, Chonnam National University, Chonnam, Republic of Korea; Wuhan Botanical Garden of Chinese Academy of Sciences, CHINA

## Abstract

In soybean, flavonoid 3′5′-hydroxylase (F3′5′H) and dihydroflavonol-4-reductase (DFR) play a crucial role in the production of anthocyanin pigments. Loss-of-function of the *W1* locus, which encodes the former, or *W3* and *W4*, which encode the latter, always produces white flowers. In this study, we searched for new genetic components responsible for the production of white flowers in soybean and isolated four white-flowered mutant lines, i.e., two *Glycine soja* accessions (CW12700 and CW13381) and two EMS-induced mutants of *Glycine max* (PE1837 and PE636). *F3′5′H* expression in CW12700, PE1837, and PE636 was normal, whereas that in CW13381 was aberrant and missing the third exon. Sequence analysis of *F3′5′H* of CW13381 revealed the presence of an indel (~90-bp AT-repeat) in the second intron. In addition, the *F3′5′H* of CW12700, PE1837, and PE636 harbored unique single-nucleotide substitutions. The single nucleotide polymorphisms resulted in substitutions of amino acid residues located in or near the SRS4 domain of F3′5′H, which is essential for substrate recognition. 3D structure modeling of F3′5′H indicated that the substitutions could interfere with an interaction between the substrate and heme group and compromise the conformation of the active site of F3′5′H. Recombination analysis revealed a tight correlation between all of the mutant alleles at the *W1* locus and white flower color. On the basis of the characterization of the new mutant alleles, we discussed the biological implications of *F3′5′H* and *DFR* in the determination of flower colors in soybean.

## Introduction

Soybean [*Glycine max* (L.) Merr.] has six different loci (i.e., *W1*, *W2*, *W3*, *W4*, *Wm*, and *Wp*) that control flower color. The range of flower colors includes dark purple, purple, light purple, pink, magenta, near-white, and white [1,2]. *W1*, *W3*, *W4*, *Wm*, and *Wp* encode flavonoid 3′5′-hydroxylase (F3′5′H), dihydroflavonol-4-reductase 1 (DFR1), DFR2, flavonol synthase, and flavanone 3-hydroxylase (F3H), respectively; *W2* corresponds to an MYB transcription factor that regulates the pH of petal vacuolar sap [3–11]. Among the various flower colors, purple and white prevail by 66.8% and 32.3%, respectively, according to the United States Department of Agriculture-Germplasm Resource Information Network (USDA-GRIN; http://www.ars-grin.gov/) [2].

**Fig 1:**
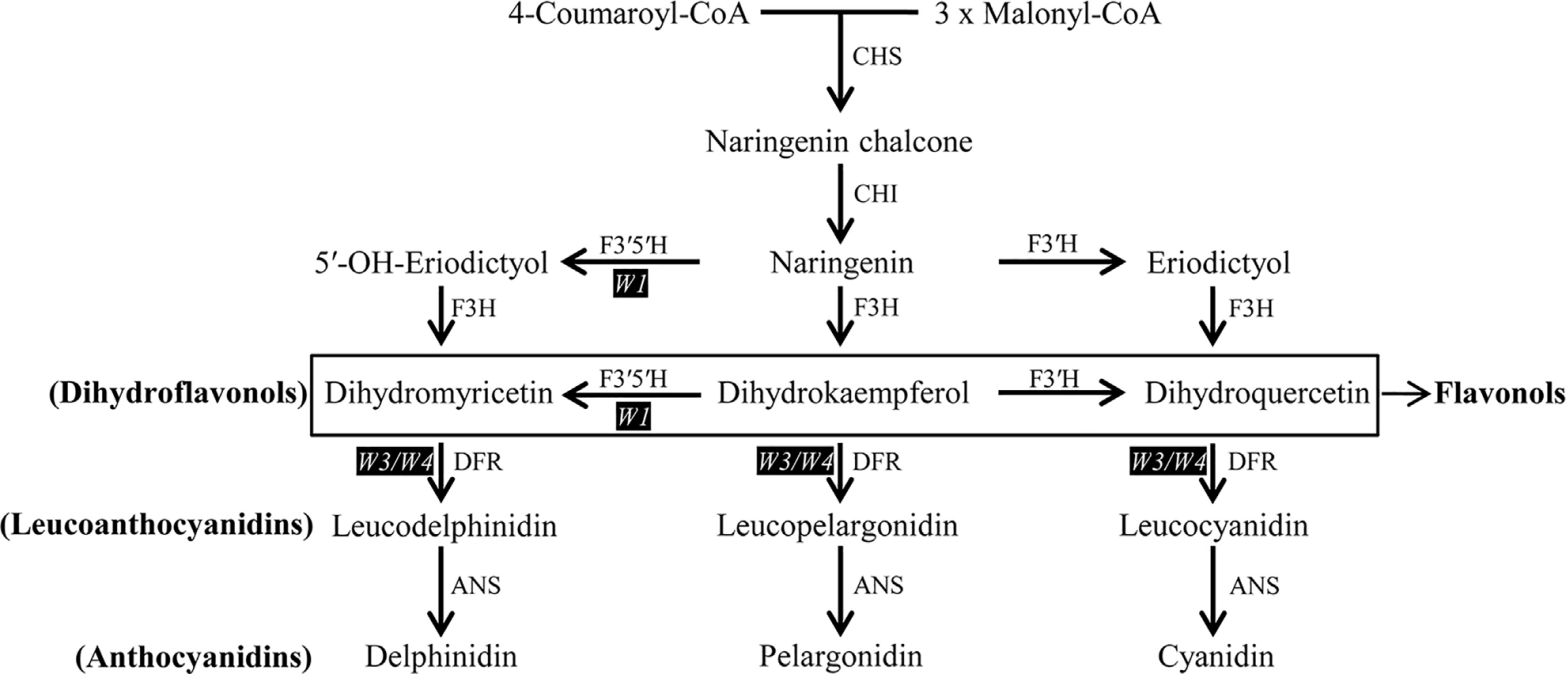
Schematic representation of the simplified biosynthesis pathway for anthocyanins. Gene loci are indicated in white letters with a black highlight. ANS, anthocyanin synthase; CHS, chalcone synthase; CHI, chalcone isomerase; DFR, dihydroflavonol-4-reductase; F3H, flavanone 3-hydroxylase; F3′H, flavonoid 3′-hydroxylase; F3′5′H, flavonoid 3′5′-hydroxylase.

In soybean, F3′5′H and DFR are essential enzymes for the synthesis of anthocyanin pigments (purple) in the flavonoid biosynthesis pathway (Fig 1) [12]. F3′5′H plays a crucial role in the determination of flower color (purple/blue) in plants, such as those that belong to the genera *Glycine*, *Pisum*, *Petunia*, *Ipomoea*, *Gentiana*, and *Vitis*. F3′5′H is a heme (Fe^3+^)-containing cytochrome P450 enzyme that hydroxylates 3′ and 5′ positions of the β ring of naringenin or dihydrokaempferol, subsequently leading to the formation of delphinidin-based anthocyanin pigments [4,13] (Fig 1). Loss-of-function mutations of *F3′5′H* caused by insertions of different transposable elements leads to the loss of delphinidin-based production of anthocyanins and resulted in changes in flower color from blue to pink, as described in such mutants in a hybrid of *Petunia* [14,15] and *Gentiana scabra* [16]. However, the anthocyanin biosynthesis pathway in legumes, at least in soybean, might be different from that in other plant species [17]. In soybean, mutations in *F3′5′H* are associated with white flowers [5]. Dominant and recessive alleles of the *W1* locus, which encodes F3′5′H, display purple and white flower color, respectively [1]. White flower color in Williams 82 (*G*. *max*) is due to the 65-bp insertion and 12-bp deletion in the coding region of *F3′5′H*, which results in premature translation and thus non-functional F3′5′H proteins [5]. Park et al. [18] reported that *F3′5′H* of all 99 landraces with white flowers has the same gene sequence as that of Williams 82.

In contrast to the flower color of cultivated soybeans, the color of flowers of the wild soybean accessions [*Glycine soja* Sieb. and Zucc.] in the USDA-GRIN collection is almost purple. However, a white-flowered *G*. *soja* line (PI 424008C) was found among the progeny of a purple-flowered *G*. *soja* accession (PI 424008A) that originated from South Korea [19]. On the basis of DNA marker analysis and the color phenotype of PI 424008C, Chen and Nelson [19] presumed that a mutation may have occurred at the *W1* locus during propagation at USDA. However, the molecular nature of the mutation remains unknown to date. A mutable accession (B00146-m) with variegated purple-white flowers was isolated from *G*. *soja* accessions in Russia [20]. The variegated flowers in the mutable accession was due to the insertion of an active transposon (*Tgs1*) in the first exon of *F3′5′H*. Excision of the active transposon resulted in revertant (purple, B00146-r) and mutant (white, B00146-w) lines. It was confirmed that B00146-w had an insertion of two nucleotides (CA) in the first exon of *F3′5′H*, which was due to the transposon footprint and resulted in a truncated version of F3′5′H and white flowers [20].

The DFR enzyme catalyzes the production of leucoanthocyanins, which are converted to anthocyanins in the ensuing step of the anthocyanin biosynthesis pathway (Fig 1). The relationship between DFR and white flower mutants has been studied in many different plant species. Repression of *DFR* expression in *Dendrobium* Sonia (an orchid) resulted in white tissue in place of purple [21]. The wild-type pink flowers of *Nicotiana tabacum* were replaced with white ones in a DFR-deficient mutant [22]. In soybean, DFR-encoding genes co-segregated with two genetic loci, namely, *W3* (*DFR1*) and *W4* (*DFR2*), and they were epistatic to each other under the *W1* allelic background [10,11]. Double mutations in *W3* and *W4*, i.e., *w3 w4*, led to the development of near-white flowers in soybean [11,23,24].

Loss-of-function mutations in *W1* or *w3 w4* double mutations always result in white or near-white flowers, respectively, in soybean. This study was undertaken to search for new soybean mutants that produce white flowers. The lines with white flowers (PE1837 and PE636) were isolated from a mutagenized population of the cultivar Pungsannamul, which normally produces purple flowers. In addition, *G*. *soja* accessions with white flowers (CW12700 and CW13381) were analyzed.

## Results

### Segregation of White-Flowered Soybean Mutants

**Fig 2:**
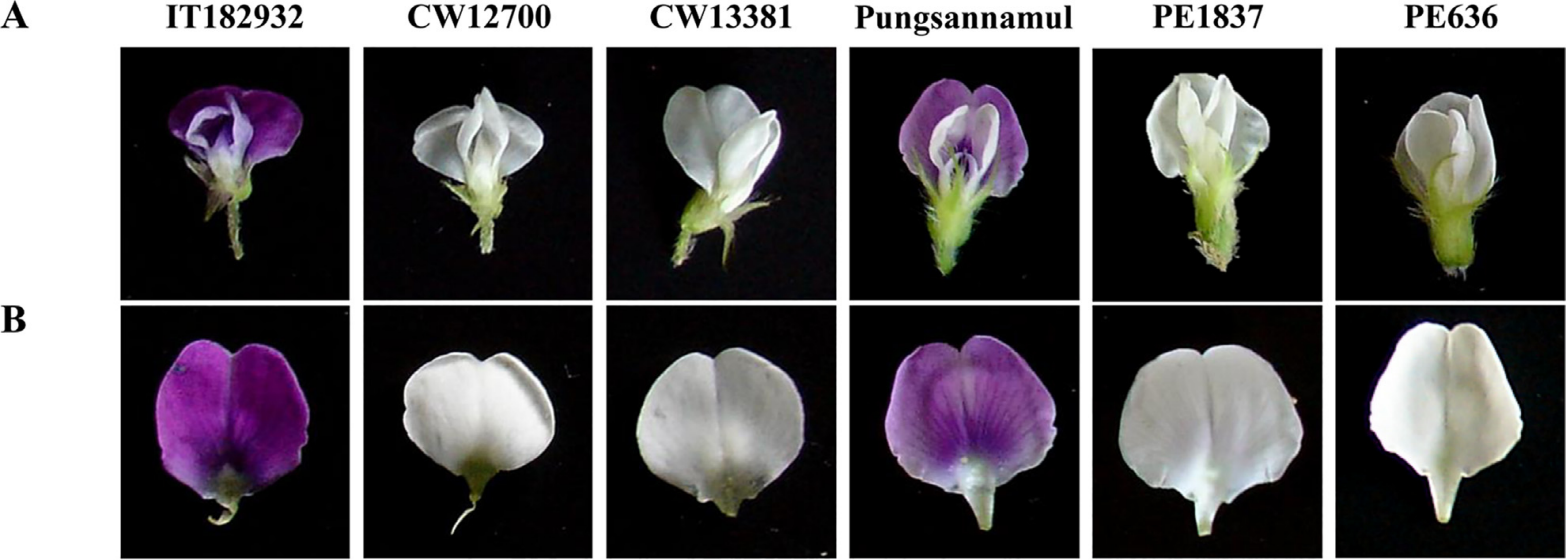
Photographic images showing flower colors of the plant materials. IT182932 (purple flower), CW12700 (white), and CW13381 (white) are *Glycine soja* accessions. EMS mutant lines, PE1837 (white) and PE636 (white), were isolated from the progeny of *Glycine max* ‘Pungsannamul’ (purple) treated with EMS. (A) A whole flower. (B) A banner petal.

In this study, wild soybean accessions (CW12700 and CW13381) and two ethyl methanesulfonate (EMS)-induced mutant lines (PE1837 and PE636), all of which produce white flowers, were isolated (Fig 2). For the segregation analysis, crosses between the two white-flowered *G*. *soja* lines and IT182932 and between the two white-flowered EMS lines and Pungsannamul were performed (Table 1). A total of 64 plants of the F_2_ population from the cross between CW12700 and IT182932 segregated into 45 plants with purple flowers and 19 plants with white flowers. The segregation fitted a 3:1 ratio (Table 1). Similar patterns of segregations were observed in the F_2_ progenies of the other crosses (CW13381 × IT182932; PE1837 × Pungsannamul; PE636 × Pungsannamul) (Table 1). The segregation analysis suggests that a single recessive gene controls the white flower color of each mutant.

**Table 1:** Segregation and co-segregation analyses.

**Cross**	No. of F_2_ plants	**Phenotype**					**Genotype**					
		**Purple**	**White**	**Expected**	χ^2^	*p* value†	A*	H*	B*	**Expected**	χ^2^	*p* value†
CW12700 x IT182932	64	45	19	3:1	0.7500	0.3865	16	29	19	1:2:1	0.8438	0.6558
CW13381 x IT182932	103	75	28	3:1	0.2621	0.6087	30	46	27	1:2:1	1.3495	0.5093
PE1837 x Pungsannamul	112	84	28	3:1	0.0270	0.8690	31	53	28	1:2:1	0.5130	0.4730
PE636 x Pungsannamul	114	84	30	3:1	0.0270	0.8690	26	59	29	1:2:1	0.2980	0.8610

**†**Not significant (*p* value >0.05)

* A, wild-type F_2_ plants with purple flowers; H, heterozygous F_2_ plants with purple flowers; B, homozygous F_2_ plants with white flowers

### Expression Profiling of Flower Color Loci

**Fig 3:**
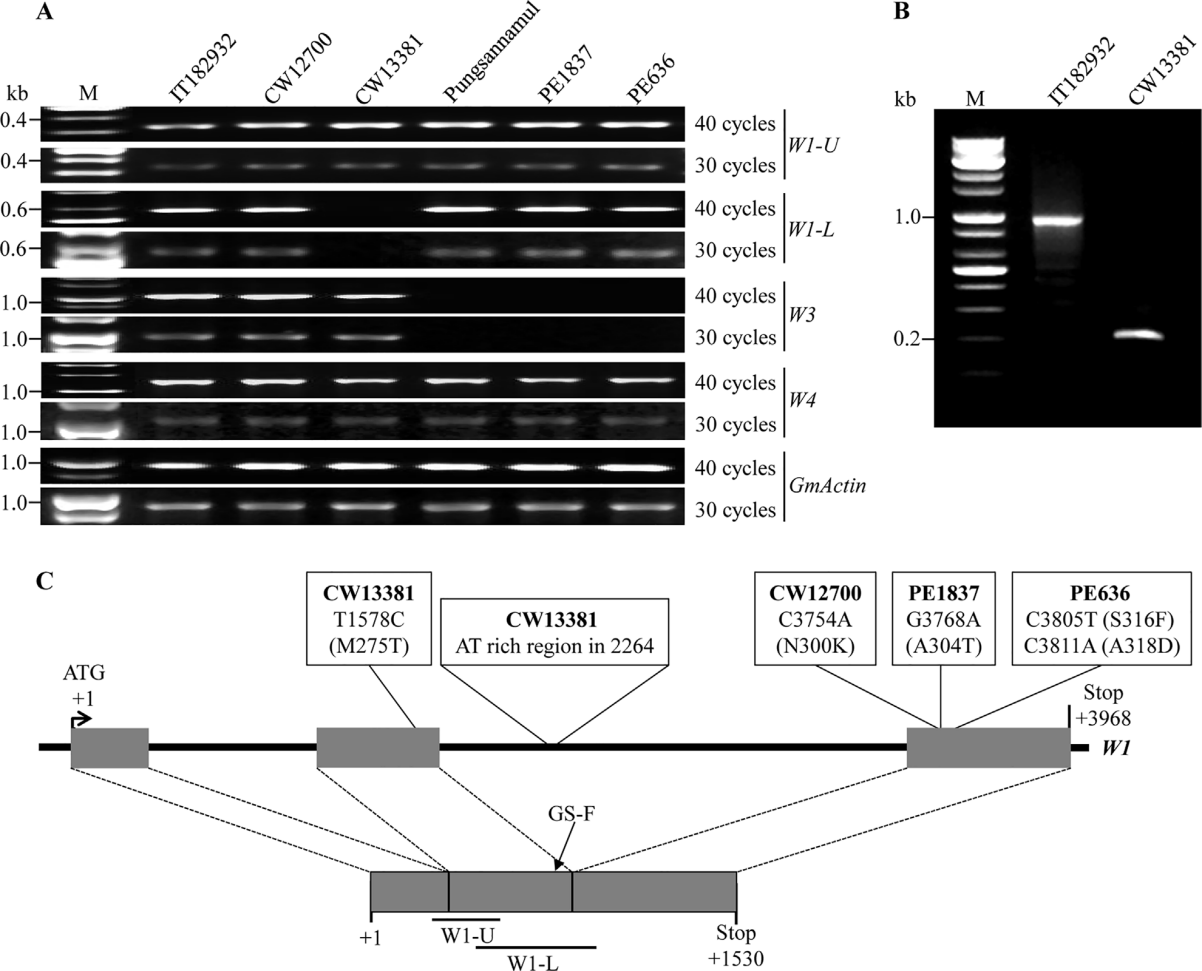
Expression profiles of *W1*, *W3*, and *W4* and schematic representation of different *F3′5′H* alleles from Pungsannamul. (A) RT-PCR analysis of *W1*, *W3*, and *W4* in purple-flowered lines (IT182932 and Pungsannamul) and white-flowered lines (CW12700, CW13381, PE1837, and PE636). *GmActin*, a housekeeping gene, was used as the loading control. M, a 1-kb molecular marker. (B) 3′-Rapid amplification of cDNA ends (RACE) analysis of the *W1* locus from IT182932 and CW13381. (C) Mutational changes in the recessive alleles at the *W1* locus are indicated in white boxes. Exons are indicated in grey boxes. *W1-U* and *W1-L* denote the 5′ and 3′ parts of *W1*, respectively. GS-F was used in 3′-RACE analysis as the gene specific forward primer.

In soybean, white or near-white flower color develops because of either a single recessive allele (*w1*) at the *W1* locus [5] or double recessive alleles (*w3 w4*) at *W3* and *W4* under the *W1* allelic background [11, 23]. However, it is practically difficult to distinguish between white and near-white flowers in the field because of certain environmental conditions [25]. Therefore, to examine whether the above-mentioned mutations (CW12700, CW13381, PE1837, and PE636) overlap with previously identified flower color loci, we determined the expression levels of *W1*, *W3*, and *W4* by using RT-PCR analysis (Fig 3A).

With respect to the *W4* locus, *DFR2* showed similar levels of expression (1175 bp in size) in the all accessions examined, indicating that all the four mutants have normal *DFR2* expression. With respect to the *W3* locus, *DFR1* also displayed similar levels of expression (1092 bp in size) in all the *G*. *soja* accessions, namely, IT182932, CW12700, and CW13381. However, no *DFR1* expression was detected in the two EMS lines (PE1837 and PE636) and in Pungsannamul. We previously reported that Pungsannamul has a *w3* recessive allele with nullified expression of *DFR1* [24]. Therefore, the results indicate that expression profiles of both *W3* and *W4* of all the four mutants are similar to those of their corresponding parental lines.

To determine the expression level of *W1*, we performed RT-PCR analysis of 5′- and 3′-half regions of *F3′5′H* (*W1-U* and *W1-L*, respectively; Fig 3A). *W1-U* products amplified from all the accessions were 331 bp in size, and their levels were not different from each other. Similarly, *W1-L* products from all the accessions, except CW13381, were 558 bp in size, and their levels were similar to each other. CW13381 did not produce *W1-L*, indicating that CW13381 has an aberrant allele of *F3′5′H*.

To identify the molecular nature that causes the aberrancy of *F3′5′H* expression in CW13381, we performed 3′-rapid amplification of cDNA ends (RACE) analysis by using a gene-specific forward primer (GS-F, 5′-TCACAAGAGAAAGGGCAAGC-3′) that corresponds to the second exon of *F3′5′H* (Fig 3B and S1 Fig). The 3′-RACE products from IT182932 were ~1 kb in size and contained the second and third exons as well as a 188 bp 3′-untranslated region (GenBank accession number, KX077981). However, a 3′-RACE product from CW13381 was ~0.2 kb in size and had only the second exon and additional 61 bp nucleotides from the second intron of *F3′5′H*. The result indicates that *F3′5′H* of CW13381 may have a defect in splicing or transcription.

### Analysis of Nucleotide Sequences of *DFR2* and *F3′5′H*

To determine the involvement of *W1* and *W4* in the allelic variations of the four mutants, we analyzed the genomic sequences of *F3′5′H* and *DFR2*. First, the genomic sequences of *DFR2* (nucleotide position, -4 to 3416) from IT182932, Pungsannamul, and three mutants (CW12700, PE1837, and PE636) were analyzed. *DFR2* sequences of the EMS mutants, PE1837 and PE636, had no polymorphisms when compared with those of Pungsannamul (GenBank accession number, KU376490). In contrast, *DFR2* of CW12700 (GenBank accession number, KX077985) showed many polymorphisms in its introns when compared with that of IT182932 (GenBank accession number, KX077986). In addition, *DFR2* of CW12700 also had two single nucleotide polymorphisms (SNPs) in its sixth exon (T2894A and G2939A), consequently substituting Glu for Val and Gln for Arg at amino acid positions 338 and 353, respectively. However, those alterations in amino acid sequences have been reported to be present in functional DFR2 proteins of the *G*. *max W4* alleles, indicating that the changes may not affect the DFR2 protein function. Further, sequence analysis of the *W4* locus showed that both IT182932 and CW12700 had a deletion of 367-bp nucleotides in the third intron of *DFR2*, when compared with that of *G*. *max* (S2 Fig).

Nucleotide sequences of *F3′5′H* from all the four mutants were also analyzed (from -64 to 4534, where +1 corresponds to the first nucleotide of the start codon; Fig 3C). When compared with *F3′5′H* of Pungsannamul (GenBank accession number, KU376489), that of PE1837 has a single-nucleotide substitution (G to A) at nucleotide position 3768 in the third exon, resulting in the substitution of Thr for Ala at amino acid position 304. *F3′5′H* of PE636 has two single-nucleotide substitutions at nucleotide positions 3805 (C to T) and 3811 (C to A) in the third exon, substituting Phe for Ser and Asp for Ala at amino acid positions 316 and 318, respectively. With the exception of those SNPs, *F3′5′H* sequences of both PE1837 and PE636 were identical to that of Pungsannamul.

When the *F3′5′H* sequence of CW12700 (GenBank accession number, KX077983) was compared with that of IT182932 (GenBank accession number, KX077984), an SNP (C to A) was detected at nucleotide position 3754 in the third exon, substituting Lys for Asn at amino acid position 300 of F3′5′H (Fig 3C and S3 Fig). In addition, when the *F3′5′H* sequence of CW13381 (GenBank accession number, KX077982) was compared with those of IT182932 and CW12700, a distinctive difference was the presence of an indel (a ~90-bp AT-repeat) in the second intron (Fig 3C and S3 Fig). An SNP in the second exon was also detected (T to C) at nucleotide position 1578, resulting in the substitution of Met to Thr (at amino acid position 275) in the non-conserved region of F3′5′H (Fig 3C). The precise length of the AT-run indel could not be determined because of the replication slippage that occurs during sequencing. The AT-run indel may be a primary cause for the aforementioned aberrancy of *F3′5′H* expression, probably leading to aberrant splicing or transcription. Several additional polymorphisms were detected in introns of *F3′5′H* from CW12700 and CW13381 when compared with that of IT182932. The new mutant alleles of *G*. *soja* (CW12700 and CW13381) were dubbed *w1-s1* and *w1-s2*, respectively, and the two new EMS mutant alleles (PE1837 and PE636), *w1-p1* and *w1-p2*, respectively.

**Fig 4:**
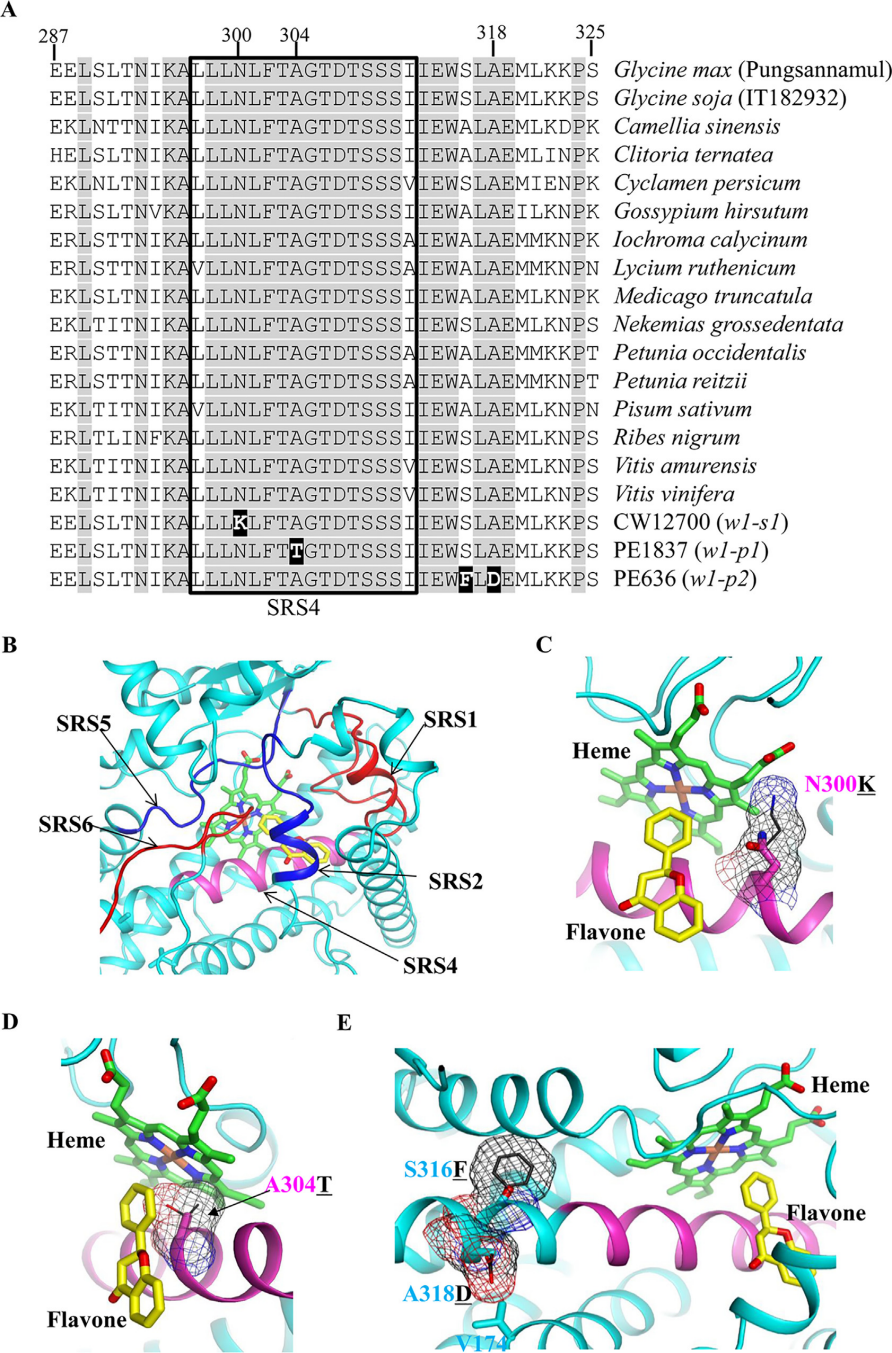
Amino acid alignment of F3′5′H proteins and 3D-modeling simulation of the effect of amino acid substitutions on structures of F3′5′H. (A) GenBank accession numbers of F3′5′H proteins are as follows: *Camellia sinensis*, AAY23287; *Clitoria ternatea*, BAF49293; *Cyclamen persicum*, ACX37698; *Gossypium hirsutum*, ACH56524; *Iochroma calycinum*, AIY22750; *Lycium ruthenicum*, AGT57963; *Medicago truncatula*, XP_013459330; *Nekemias grossedentata*, AGO02173; *Petunia occidentalis*, BAF34571; *Petunia reitzii*, BAF34572; *Pisum sativum*, ADW66160; *Ribes nigrum*, AGI16385; *Vitis amurensis*, ACN38269; and *Vitis vinifera*, BAE47007. Identical amino acids are in grey. Relevant amino acid substitutions are indicated in white letters with a black highlight. The thick black box indicates the SRS4 domain. N (Asn) → K (Lys) in *w1-s1*; A (Ala) → T (Thr) in *w1-p1*; and S (Ser) → F (Phe), and A (Ala) → D (Asp) in *w1-p2*. (B) 3D-model structure of the F3′5′H active site consisting of the SRS domains, a heme (green), and a flavone (yellow). (C) 3D-model structure showing the N300K substitution in CW12700 (*w1-s1*). The Asn300 residue is shown in the magenta stick, and the Lys300 substitute is overlaid by the black line and mesh. (D) 3D-model structure showing the A304T substitution in PE1837 (*w1-p1*). The Ala304 residue is shown in the magenta stick, and the Thr304 substitute is represented by the black line and mesh. (E) 3D-model structure showing the S316F and A318D substitutions in PE636 (*w1-p2*). The Ser316 and Ala318 residues are shown in cyan sticks, and the Phe316 and Asp318 substitutes are overlaid by black lines and meshes.

The six functional substrate recognition sites (SRS) conserved in F3′5′H enzymes are essential for substrate specificity and 3′5′-hydroxylase activities [26,27]. To infer the effect of the amino acid substitutions on the activity of F3′5′H proteins produced from the three recessive alleles (*w1-s1*, *w1-p1*, and *w1-p2*), we performed amino acid alignment of F3′5′H proteins from 15 different plant species (Fig 4A). The single amino acid substitutions in F3′5′H proteins of *w1-s1* (Lys for Asn at position 300) and *w1-p1* (Thr for Ala at position 304) mutants occurred in the highly conserved SRS4 domain. In the *w1-p2* mutant, two different single amino acid substitutions lie adjacent to the SRS4 domain. It should be noted that the substitution of acidic Asp (D) for aliphatic Ala (A) at position 318 of *w1-p2* is located in the highly conserved region of the cytochrome P450 superfamily. In summary, F3′5′H proteins of *w1-s1*, *w1-p1*, and *w1-p2* alleles have critical substitutions in/near the SRS4 domain, raising the possibility that those substitutions may disrupt the function of F3′5′H proteins.

To obtain further insight into the effect of the amino acid substitutions in F3′5′H proteins on substrate binding, we performed modeling of F3′5′H by using a web-based 3D structure modeling program, Phyre2 [28]. The predicted 3D model of F3′5′H is most similar to that of human P450 1A2 (PDP id: 2HI4) [29]. Therefore, based on the positions of a heme and α-naphthoflavone shown in the human P450 1A2 structure, both the heme and flavone were modeled in the F3′5′H structure by superposition (Fig 4B–4E). The 3D model showed that the heme group of F3′5′H responsible for the catalytic reactivity is surrounded by the indicated SRS domains, as observed in other plant P450 enzymes [30,31]. On the basis of the prediction that the enzymatic activity of F3′5′H proteins might be lost in *w1-s1* (Asn300Lys), *w1-p1* (Ala304Thr), and *w1-p2* (Ser316Phe and Ala318Asp), we modeled each amino acid substitution. First, the Asn residue is located between the heme and flavone groups (Fig 4C). When Asn is mutated to Lys in *w1-s1*, the ε-amino group of Lys can extend to the proximate environment of the heme group. Besides, disappearance of the carboxamide group because of the substitution of Asn might affect the stability of the flavone as well. Therefore, the substitution of Asn for Lys could probably affect the flavone-hydroxylation activity. Second, the position of Ala is between the heme and a phenyl ring of the flavone (Fig 4D). If Ala is substituted with Thr in *w1-p1*, the hydroxyl group of Thr could inhibit the proper binding of the flavone substrate. Third, although the Ser and Ala residues are not located in the SRS4 domain, they lie in the same helix containing the SRS4 domain (Fig 4E). The aromatic Phe and acidic Asp substitutes in *w1-p2* may probably affect the conformation of the active site by perturbing the SRS4-containing helix. Taken together, all the amino acid substitutions could lead to loss-of-function of the F3′5′H enzyme.

### Co-Segregation of the White Flower Phenotype with Soybean Mutants

**Fig 5:**
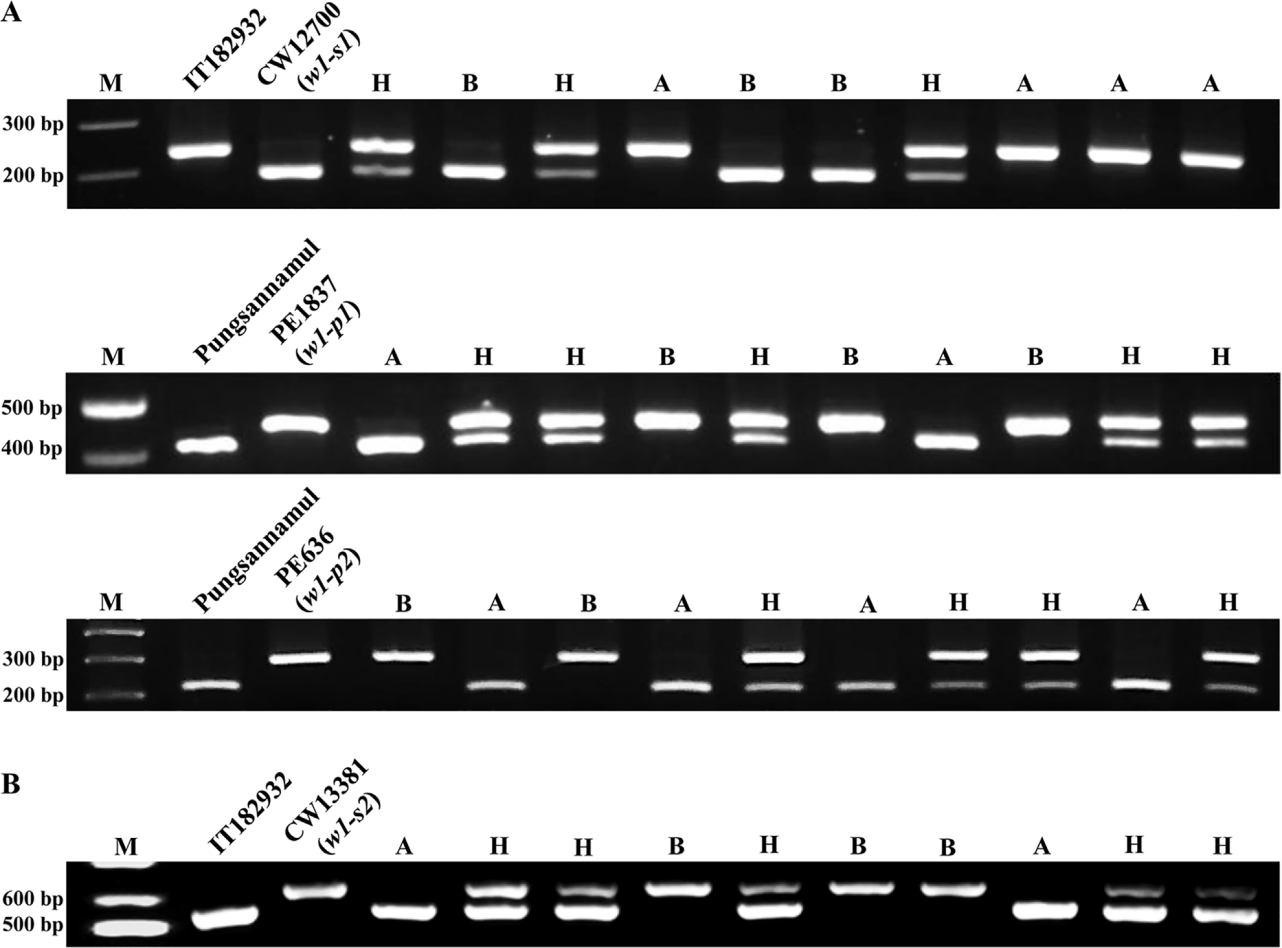
Derived cleaved amplified polymorphic sequence (dCAPS) analysis of CW12700 (*w1-s1*), PE1837 (*w1-p1*), and PE636 (*w1-p2*) and indel analysis of CW13381 (*w1-s2*). (A) dCAPS analysis of F_2_ populations derived from the breeding crosses, such as CW12700 × IT182932, PE1837 × Pungsannamul, and PE636 × Pungsannamul. The PCR products were digested by *Spe*I, *Pst*I or *Hae*III. (B) Indel analysis of the F_2_ population derived from a cross between CW13381 and IT182932. M, a 1-kb molecular marker; H, heterozygous F_2_ plants with purple flowers; A, wild-type F_2_ plants with purple flowers; and B, homozygous F_2_ plants with white flowers.

Derived cleaved amplified polymorphic sequence (dCAPS) analyses were performed to determine the co-segregation of the *w1-s1*, *w1-p1*, and *w1-p2* alleles with the white color phenotype. A set of dCAPS primers amplified 240-bp PCR products from both CW12700 (*w1-s1*) and IT182932 (*W1*). However, PCR products from *w1-s1* were digested by *Spe*I and cut to produce a 194-bp fragment, whereas those from *W1* remained uncut. The dCAPS markers co-segregated with flower colors of the F_2_ plants derived from a cross between CW12700 and IT182932: F_2_ plants with white flowers displayed only the shorter fragment, whereas F_2_ plants with purple flowers had only the longer fragment or both (Fig 5A).

In the dCAPS analysis of PE1837 (*w1-p1*) and Pungsannamul (*W1*), PCR products from the former (476 bp in size) remained uncut by *Pst*I, whereas those from the latter were cut to a 431-bp fragment. In the dCAPS analysis of PE636 (*w1-p2*) and Pungsannamul, PCR products from the former (286 bp in size) remained uncut by *Hae*III, whereas those from the latter were cut to generate a 231-bp fragment. Similar patterns of co-segregation were observed in the dCAPS analysis of the F_2_ populations from PE1837 × Pungsannamul and PE636 × Pungsannamul. F_2_ populations from all of the crosses showed segregation ratios that were consistent with the expected 1:2:1 ratio (Table 1).

Indel analysis of F_2_ individuals derived from a cross between CW13381 and IT182932 showed that *w1-s2* had a longer fragment (~650 bp) than *W1* (~560 bp) because of the presence of the AT-run indel (Fig 5B). The markers co-segregated with flower colors of the F_2_ plants. The segregation pattern was consistent with the expected 1:2:1 ratio (Table 1).

## Discussion

In this study, we isolated four new white-flowered mutant lines (CW12700, CW13381, PE1837, and PE636). Segregation and co-segregation analyses showed that the single recessive alleles of the *W1* locus resulted in the white flower color. Sequence analysis of the *W1* locus from CW12700, PE1837, and PE636 revealed that they harbored unique SNPs that led to single amino acid substitutions, i.e., Asn300Lys in *w1-s1*, Ala304Thr in *w1-p1*, and Ser316Phe/Ala318Asp in *w1-p2*. These unique amino acid changes in the F3′5′H proteins occurred in or adjacent to the SRS4 domain, which is involved in contacting substrates of F3′5′H and plays an important role in constituting the active site [26,32]. 3D structure modeling of F3′5′H suggested that the amino acid substitutions in *w1-s1* and *w1-p1* lines could interfere with an interaction between the substrate and heme group, and that the substitutions in *w1-p2* could disrupt the helix containing the SRS4 domain and probably compromise the conformation of the active site of F3′5′H. Therefore, the unique amino acid changes positioned in or adjacent to the SRS4 domain might cause loss-of-function of the F3′5′H proteins. In a previous study, a *G*. *soja* accession (B09121) with the *w1-lp* recessive allele at the *W1* locus was shown to have a distinctive substitution at amino acid position 210 (Met for Val), which was just adjacent to the SRS2 domain [33]. The study suggested that the production of light purple flowers was due to a reduction in the activity of the F3′5′H proteins [33]. In case of CW13381 (*w1-s2*), the third exon of *F3′5′H* that encodes the SRS4 domain was not retained in the mRNAs, probably due to an effect of the indel (the ~90-bp AT-repeat) on splicing or transcription.

Interestingly, sequence analysis of the *W4* locus showed that the 367-bp deletion detected in *DFR2* of both IT182932 and CW12700 was identical to the one detected in the near-white *kw4* mutant, as described by Yan et al. [34]. According to that study, the *kw4* mutant showed no expression of *DFR2*, and, thus, the authors predicted that the deletion might be one of the reasons for the lack of expression of *DFR2* [34]. However, our study demonstrated that both IT182932 and CW12700 showed normal expression of *DFR2*, irrespective of the presence of the 367-bp deletion (Fig 3A). Therefore, we suggest that the deletion in the intron may not be responsible for the expression patterns of *DFR2*, and that no expression of *DFR2* in *kw4* may be due to the presence of another mutation in its 5′-upstream region [34].

In plants, there are three types of anthocyanin pigments: cyanidins (red/magenta/blue), delphinidins (purple/blue), and pelargonidins (orange/red). Production of cyanidin- and delphinidin-based anthocyanins is dependent on two key enzymes, namely, F3′H and F3′5′H, that add 1 or 2 hydroxyl groups to the β ring of dihydroquercetin or dihydromyricetin, respectively [33,34] (Fig 1). Production of pelargonidin-based anthocyanins requires neither of the key enzymes [35,36]. Plants such as petunia, tropical water lily, cymbidium, and crape myrtle (*Lagerstroemia* sp.) mostly produce delphinidins and cyanidins [37–40]. In *Eustoma grandiflorum* (Lisianthus), pink flower color is determined by pelargonidins, and pale purple flower color arises from a combination of cyanidins and pelargonidins [41,42]. Blue flower color of gentian is determined by delphinidin derivatives [43]. Likewise, major types of anthocyanins in soybean are delphinidin-based pigments (delphinidin 3,5-di-*O*-glucoside, delphinidin 3-*O*-glucoside, malvidin 3,5-di-*O*-glucoside, and petunidin 3,5-di-*O*-glucoside) [44,45]. The lack of cyanidin-based anthocyanins in soybean suggests that F3′H plays no role in anthocyanin production for flower color. In addition, soybean DFR may have a preference for dihydromyricetin, rather than dihydrokaempferol and dihydroquercetin, as a substrate, as the loss-of-function of *W3* (*DFR1*) and *W4* (*DFR2*) results in white flowers [11,23,24]. For instance, plants such as petunia and cymbidium lack varieties with orange/brick red flowers because of the absence of pelargonidin-based anthocyanins, which was consistent with the fact that dihydrokaempferol did not serve as a substrate for DFRs [37,38]. Thus, it seems that, in the anthocyanin biosynthesis pathway of soybean, the branch that produces delphinidin-based anthocyanins may be solely active. In contrast, horticultural plants such as rose, carnation, and chrysanthemum do not accumulate delphinidin-based anthocyanins because of the deficiency of F3′5′H, the key enzyme in the production of delphinidins, and lack varieties with blue/violet flowers [46]. Therefore, we suggest that loss of the F3′5′H activity in soybean always leads to no anthocyanin synthesis, consequently resulting in white flowers instead of shifting to the synthesis of other types of anthocyanins (cyanidins and pelargonidins).

## Materials and Methods

### Plant Material

The wild soybean accessions with white flowers (CW12700 and CW13381) were obtained from the Chonnam National University soybean germplasm collection. Two EMS-induced mutant lines with white flowers (PE1837 and PE636) were isolated from a population of the soybean cultivar Pungsannamul. Pungsannamul [47] and IT182932 were used in this study (Table 2 and Fig 2). For the segregation analysis, breeding crosses between the two white-flowered *G*. *soja* lines and IT182932 and between the two white-flowered EMS lines and Pungsannamul were performed. F_2_ individuals of the populations have been described in detail in Table 1. All soybean accessions were grown in the affiliated experimental fields of Kyungpook National University (Gunwi, 36°07′ N, 128°38′ E, Republic of Korea).

**Table 2:** Plant materials of *Glycine soja* and *Glycine max* used in this study.

	**Accession/Line**	**Flower color**	**Origin**
*G*. *Soja*	IT182932	Purple	RDAᵃ
	CW12700 (*w1-s1*)	White	CWLGCᵇ
	CW13381 (*w1-s2*)	White	CWLGCᵇ
*G*. *max*	IT263156	Purple	RDAᵃ (Pungsannamul)
	PE1837 (*w1-p1*)	White	EMS mutant from Pungsannamul
	PE636 (*w1-p2*)	White	EMS mutant from Pungsannamul

ᵃRDA- the plant germplasm collection at the Rural Development Administration, Republic of Korea

ᵇCWLGC- Chung’s wild legume germplasm collection at the Chonnam National University, Republic of Korea

### Isolation of RNA and cDNA Synthesis

Total RNAs were extracted from freeze-dried standard petals (200 mg) of IT182932, CW12700, CW13381, Pungsannamul, PE1837, and PE636 by using the phenol-chloroform and lithium chloride precipitation methods [48]. The extracted RNAs were treated with DNaseI to remove DNA contamination (TaKaRa, Japan). Synthesis of first-strand cDNA was performed by reverse transcription of total RNAs with an oligo-dT_(20)_ primer and Superscript III, according to the manufacturer’s instructions (Invitrogen, Carlsbad).

### RT-PCR Analysis

PCR was performed to determine the transcript levels of *W1*, *W3*, and *W4* by using the first-strand cDNA, and *GmActin* (a housekeeping gene) was used as the loading control. The PCR conditions were follows: initial denaturation at 94°C for 5 min, followed by 30 or 40 cycles of denaturation at 94°C for 20 s, annealing at 58°C for 40 s, and extension at 72°C for 1 min; a final extension was performed at 72°C for 5 min. PCR products were separated using 1.2% agarose gels, stained with ethidium bromide, visualized under UV light, and finally subjected to sequencing (Solgent, Korea). The primers used are listed in Table 3.

**Table 3:** List of primers.

Analysis	Primer name	Sequence (5′-3′)
RT-PCR	W1-UF	CTGCTCGTGCCTTCCTCAAA
	W1-UR	TCAAACACTCGACGACTCAA
	W1-LF	GAAAGGCACTTGATGATTGG
	W1-LR	TTATGCTGGGCTTCTTCAAC
	W3-F	AAATGGGTTCAGCATCCGAAA
	W3-R	AGCAAGTTGCACAGCCATCA
	W4-F	GAACATGGGTTCAAGTTCAGCA
	W4-R	TTCATCACACATGATCCCTAAAGA
*F3′5′H* sequencing	W1-F1	ACGACACCACACATCCATTT
	W1-R1	TCAAACACTCGACGACTCAA
	W1-F2	GAAAGGCACTTGATGATTGG
	W1-R2	TTGCCAAAACAAACATGCTA
	W1-F3	GGTACGTTGAGTGTATTGTTGG
	W1-R3	TTATGCTGGGCTTCTTCAAC
	W1-F4	TCCCTCGCCTCATAATCATA
	W1-R4	AATTAAACCAAAACAAGACAGC
dCAPS	w1-s1-F	AGTGCAAATTCAACGAATCAA
	w1-s1-R	AGGACCACTCTATTATACTTGAAGATGTATCGGTGCCTGCGGTGACTAG
	w1-p1-F	TTTTGTTCTAACAACTATATATGCTATTTTGTTCCAGAACCTATTCACT
	w1-p1-R	AAATCCTCCTCCCAGCACCA
	w1-p2-F	TTCACCGCAGGCACCGATACATCTTCAAGTATAATAGAGTGGTCCTTGG
	w1-p2-R	TTCAGCCTAGTGTTCTCGGG
Indel	w1-s2-F	AGCCTTCTGCATATGTCGTC
	w1-s2-R	ACCACAGAACAAGTGAGACTCA

### 3′-RACE PCR

For 3′-RACE, single-strand cDNAs were synthesized from 10 μg of total RNAs obtained from the petals by using a 3′-RACE adapter primer (5′-GTAATACGACTCACTATAGGGC[T]_18_). PCR for 3′-RACE was performed with gene-specific (GS-F, 5′-TCACAAGAGAAAGGGCAAGC-3′) and 3′ Nest-1 primers (5′-GTAATACGACTCACTATAGGGC-3′), according to the manufacturer’s instructions, at 94°C for 30 s, 56°C for 1 min, and 72°C for 1 min. Nested PCR was performed with gene-specific and 3′ Nest-2 primers (5′-ACGACTCACTATAGGGCTTTTT-3′) for 40 cycles of 94°C for 30 s, 56°C for 1 min, and 72°C for 1 min. The final PCR product was subjected to sequencing (Solgent, Korea).

### Genomic DNA Isolation and Sequence Analysis

Genomic DNAs from the soybean lines were isolated from trifoliate leaves by using the CTAB method [49]. Exons and introns of *F3′5′H* were amplified using PCR under the same conditions as mentioned above. The primers used for sequencing are listed in Table 3.

### dCAPS Analysis

Genomic DNAs were isolated from the F_2_ populations derived from a cross between CW12700 and IT182932 and between the two EMS lines (PE1837 and PE636) and Pungsannamul and then used for dCAPS analysis. PCR primers (Table 3) were designed to detect a single-base substitution in the *w1-s1*, *w1-p1*, and *w1-p2* alleles. A mismatch nucleotide was incorporated into each dCAPS primer to generate restriction sites (*w1-s1*, *Spe*I; *w1-p1*, *Pst*I; *w1-p2*, *Hae*III) in the amplified PCR product (S4 Fig). PCR conditions were the same as those mentioned above.

### Indel Analysis

Indel analysis of the AT-run repeat in *F3′5′H* of the *w1-s2* allele was performed to distinguish between *W1* and *w1-s2* alleles. The primers are listed in Table 3, and PCR profiles were the same as those mentioned above. The PCR products from the *w1-s2* allele were longer than those from the *W1* allele because of the existence of the AT repeat in the second intron.

## Supporting Information

S1 Fig Sequences of the 3′-RACE products from CW13381. (A) Nucleotide sequences of 3′-RACE products from CW13381 (*w1-s2*) shows the second exon and a 5′ part of the second intron of *F3′5′H*. Uppercases with grey highlights indicate the second exon; lowercases, the second intron; red, a gene-specific primer (GS-F) used for 3′-RACE. (B) A part of the chromatogram from the *w1-s2* allele. The black box indicates the poly (A) tail following the 5′ part of the second intron.
(DOCX)

S2 Fig Alignment of *DFR2* genomic sequences from Clark, *kw4*, IT182932, and CW12700 (*w1-s1*). A 367-bp deletion in the second intron is shown using the dotted line. Single-nucleotide polymorphisms present in the introns are in grey. The coding region is in bolded uppercases.
(RTF)

S3 Fig Alignment of *F3′5′H* genomic sequences from IT182932, *w1-s1*, and *w1-s2*. A single-nucleotide polymorphism detected in the third exon is in black. Other polymorphisms detected in the introns and second exon are in grey. The coding region is in uppercase; the start codon (ATG) and stop codons (TAA/TAG) are underlined.
(DOCX)

S4 Fig Schematic diagrams of dCAPS analysis to detect a SNPs in *w1-s1*, *w1-p1* and *w1-p2*. **(A)** Diagram of dCAPS analysis to detect a SNP in the *w1-s1* allele (highlighted in black). The *Spe*I site (ACTAGT) underlined was artificially introduced in the PCR products of the *w1-s1* allele by using a reverse primer with a mismatched base (C, highlighted in grey). The PCR products from IT182932 are 240-bp in length and not digested with *Spe*I, whereas those from *w1-s1* are 194-bp in length after digestion. **(B)** Diagram of dCAPS analysis to detect a SNP in the *w1-p1* allele (highlighted in black). The *Pst*I site (CTGCAG) underlined was artificially introduced in the PCR products of the *W1* allele by using a forward primer with a mismatched base (T, highlighted in grey). The PCR products from PE1837 (*w1-p1*) are 476-bp in length and not digested with *Pst*I, whereas those from *W1* are 431-bp in length after digestion. **(C)** Diagram of dCAPS analysis to detect a SNP in the *w1-p2* allele (highlighted in black). The *Hae*III site (GGCC) underlined was artificially introduced in the PCR products of the *W1* allele by using a forward primer with a mismatched base (G, highlighted in grey). The PCR products from PE636 (*w1-p2*) are 286-bp in length and not digested with *Hae*III, whereas those from *W1* are 231-bp in length after digestion.
(PDF)
