# Supplementary material for: Identification and Molecular Analysis of Four New Alleles at the W1 Locus Associated with Flower Color in Soybean
Source: PLoS One. 2016 Jul 21;11(7):e0159865. doi: 10.1371/journal.pone.0159865 (PMC4956318; doi:10.1371/journal.pone.0159865)
Supplement: S1 Fig — (A) Nucleotide sequences of 3′-RACE products from CW13381 (w1-s2) shows the second exon and a 5′ part of the second intron of F3′5′H. Uppercases with grey highlights indicate the second exon; lowercases, the second intron; red, a gene-specific primer (GS-F) used for 3′-RACE. (B) A part of the chromatogram from the w1-s2 allele. The black box indicates the poly (A) tail following the 5′ part of the second intron. (DOCX) [file pone.0159865.s001.docx]

**A**

1363 TCACAAGAGAAAGGGCAAGCCCGATTTCTTAGACACGGTAATGGCTCATCATAGTGAGAA

1423 CTCCGATGGGGAGGAACTATCGCTCACCAACATCAAGGCACTACTCTTGgtataacgctt

1483 tttatcttacttctcaaatgtgtcattttctttcttcatttttattagac

**B**


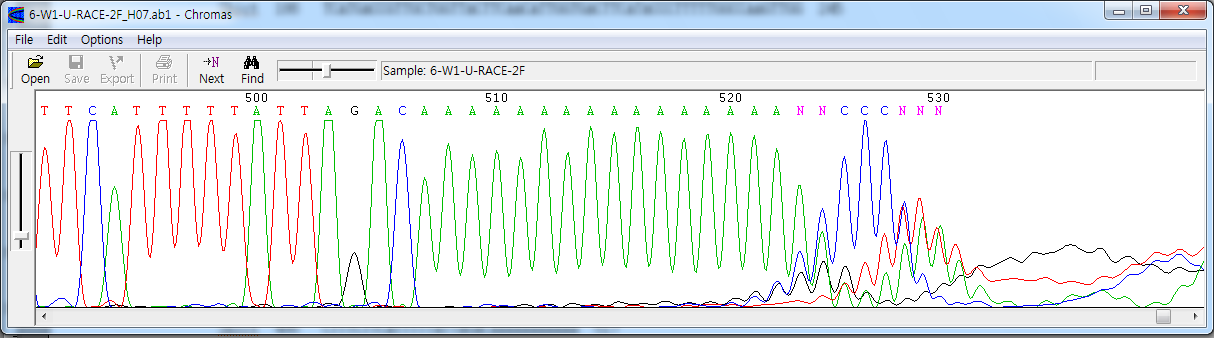


**S1 Fig. Sequences of the 3′-RACE products from CW13381.**
