# Supplementary material for: Identification and Molecular Analysis of Four New Alleles at the W1 Locus Associated with Flower Color in Soybean
Source: PLoS One. 2016 Jul 21;11(7):e0159865. doi: 10.1371/journal.pone.0159865 (PMC4956318; doi:10.1371/journal.pone.0159865)
Supplement: S2 Fig — A 367-bp deletion in the second intron is shown using the dotted line. Single-nucleotide polymorphisms present in the introns are in grey. The coding region is in bolded uppercases. (RTF) [file pone.0159865.s002.rtf]

                                               Exon 3
1586 ACCCAAACCCTGTCATCGATGAGAACTGTTGGAGCGATGTTGACTTCTGCACAAGGGTCAAAATGACTGGTTGGgttagtttttttttcttcttctttct Clark (W4)
1586 ACCCAAACCCTGTCATCGATGAGAACTGTTGGAGCGATGTTGACTTCTGCACAAGGGTCAAAATGACTGGTTGGgttagtttttttttcttcttctttct kw4 (w4)
1585 ACCCAAACCCTGTCATCGATGAGAACTGTTGGAGCGATGTTGACTTCTGCACAAGGGTCAAAATGACTGGTTGGgttagtttttttttcttcttctttct IT182932
1587 ACCCAAACCCTGTCATCGATGAGAACTGTTGGAGCGATGTTGACTTCTGCACAAGGGTCAAAATGACTGGTTGGgttagtttttttttcttcttctttct CW12700

1686 attattaaaaaatggatgaaatatgtgtttgttcatcaatgcacatagactaaatcctatggatatggaccagattgattgatttgaaattaccatggct Clark (W4)
1686 attattaaaaaatggatgaaatatgtgtttgttcatcaatgcacatagactaaatcctatggatatggaccagattgattgatttgaaattaccatggct kw4 (w4)
1685 attattaaaaaatggatgaaatatgtgtttgttcatcaatgcacatagactaaatcctatggatatggaccagattgattgatttgaaattaccatggct IT182932
1687 attattaaaaaatggatgaaatatgtgtttgttcatcaatgcacatagactaaatcctatggatatggaccagattgattgatttgaaattaccatggct CW12700

1786 gaaatcaagtttaaccgaaaatacataatcaaaaattttttacccaaaaaaaaagaattgtgtcatatatccttgtagtttttctttattcaaaaaatga Clark (W4)
1786 gaaatcaagtttatccgaaaatacataatcaaaaaaattttacccaaaaaaaa-gaattgt--catatatccttgtagtttttctttattcaa------- kw4 (w4)
1785 gaaatcaagtttatccgaaaatacataatcaaaaaaattttacccaaaaaaaa-gaattgtgtcatatatccttgtagtttttctttattcaa------- IT182932
1787 gaaatcaagtttaaccgaaaatacataatcaaaaattttttacccaaaaaaaaagaattgtgtcatatatccttgtagtttttctttattcaa------- CW12700

1886 tttctggacaatatatttgcatttcacacctagcaagataaaaaaaaaatttagtagtgattattaaaaaacatacaaaatgataagggatataatgtga Clark (W4)
1875 ---------------------------------------------------------------------------------------------------- kw4 (w4)
1876 ---------------------------------------------------------------------------------------------------- IT182932
1879 ---------------------------------------------------------------------------------------------------- CW12700

1986 tagaaaaaaataaaaaacaaaactaagtattaatgaagtgtttgtaatttaatgctatccaaatattattatttttcttcgttaacacacggccagcact Clark (W4)
1875 ---------------------------------------------------------------------------------------------------- kw4 (w4)
1876 ---------------------------------------------------------------------------------------------------- IT182932
1879 ---------------------------------------------------------------------------------------------------- CW12700

2086 tgacaaaaaccctttttttaagatagacaaggcctaacataaatgagttggggtttatgacattgtgtactatagaaagaatagagtgattatgagtttg Clark (W4)
1875 ---------------------------------------------------------------------------------------------------- kw4 (w4)
1876 ---------------------------------------------------------------------------------------------------- IT182932
1879 ---------------------------------------------------------------------------------------------------- CW12700

2186 tttcctccaagagaaaaaggaaaaacagaggagaataaataatattctcacattaattaatagtggatgaatatgattctatataatgtttccatttttt Clark (W4)
1875 ------------------------------------------------------------tagtggatgaatatgattctatataatgtttccatttttt kw4 (w4)
1876 ------------------------------------------------------------tagtggatgaatatgattctatataatgtttccatttttt IT182932
1879 ------------------------------------------------------------tagtggatgaatatgattctatataatgtttccatttttt CW12700

                                                                   Exon 4
2285 -gcagATGTATTTTGTTTCAAAGACCCTGGCGGAGCAAGAAGCGTGGAAATATGCCAAAGAGCACAACATAGACTTTATATCAGTCATTCCACCCCTTGT Clark (W4)
1915 -gcagATGTATTTTGTTTCAAAGACCCTGGCGGAGCAAGAAGCGTGGAAATATGCCAAAGAGCACAACATAGACTTTATATCAGTCATTCCACCCCTTGT kw4 (w4)
1917 tgcagATGTATTTTGTTTCAAAGACCCTGGCGGAGCAAGAAGCGTGGAAATATGCCAAAGAGCACAACATAGACTTTATATCAGTCATTCCACCCCTTGT IT182932
1919 -gcagATGTATTTTGTTTCAAAGACCCTGGCGGAGCAAGAAGCGTGGAAATATGCCAAAGAGCACAACATAGACTTTATATCAGTCATTCCACCCCTTGT CW12700


S2 Fig. Alignment of DFR2 genomic sequences from Clark, kw4, IT182932, and CW12700 (w1-s1).
