# Supplementary material for: Identification and Molecular Analysis of Four New Alleles at the W1 Locus Associated with Flower Color in Soybean
Source: PLoS One. 2016 Jul 21;11(7):e0159865. doi: 10.1371/journal.pone.0159865 (PMC4956318; doi:10.1371/journal.pone.0159865)
Supplement: S3 Fig — A single-nucleotide polymorphism detected in the third exon is in black. Other polymorphisms detected in the introns and second exon are in grey. The coding region is in uppercase; the start codon (ATG) and stop codons (TAA/TAG) are underlined. (DOCX) [file pone.0159865.s003.docx]

-64 tcatagatatcccgaatcatcaaattattacttcatagcaactagcaaattaattagcttcaccATGGACTCATTGTTACTTCTAAAAGAAATTGCCACT IT182932

-64 tcatagatatcccgaatcatcaaattattacttcatagcaactagcaaattaattagcttcaccATGGACTCATTGTTACTTCTAAAAGAAATTGCCACT *w1-s1*

-64 tcatagatatcccgaatcatcaaattattacttcatagcaactagcaaattaattagcttcaccATGGACTCATTGTTACTTCTAAAAGAAATTGCCACT *w1-s2*

37 TCCATTTTGATCTTCTTGATCACTCGTCTCTCCATTCAAACATTCCTCAAAAGCTATCGCCAGAAACTCCCACCGGGGCCAAAAGGGTGGCCAGTTGTGG IT182932

37 TCCATTTTGATCTTCTTGATCACTCGTCTCTCCATTCAAACATTCCTCAAAAGCTATCGCCAGAAACTCCCACCGGGGCCAAAAGGGTGGCCAGTTGTGG *w1-s1*

37 TCCATTTTGATCTTCTTGATCACTCGTCTCTCCATTCAAACATTCCTCAAAAGCTATCGCCAGAAACTCCCACCGGGGCCAAAAGGGTGGCCAGTTGTGG *w1-s2*

137 GTGCACTCCCTCTCATGGGAAGCATGCCTCATGTCACCTTAGCAAAGATGGCAAAAAAATATGGACCTATAATGTACCTCAAAATGGGCACTAACAACAT IT182932

137 GTGCACTCCCTCTCATGGGAAGCATGCCTCATGTCACCTTAGCAAAGATGGCAAAAAAATATGGACCTATAATGTACCTCAAAATGGGCACTAACAACAT *w1-s1*

137 GTGCACTCCCTCTCATGGGAAGCATGCCTCATGTCACCTTAGCAAAGATGGCAAAAAAATATGGACCTATAATGTACCTCAAAATGGGCACTAACAACAT *w1-s2*

237 GGTTGTGGCCTCTACTCCAGCTGCTGCTCGTGCCTTCCTCAAAACCCTTGATCAAAACTTTTCAAACCGGCCCTCCAATGCTGGTGCAACCCATTTGGCT IT182932

237 GGTTGTGGCCTCTACTCCAGCTGCTGCTCGTGCCTTCCTCAAAACCCTTGATCAAAACTTTTCAAACCGGCCCTCCAATGCTGGTGCAACCCATTTGGCT *w1-s1*

237 GGTTGTGGCCTCTACTCCAGCTGCTGCTCGTGCCTTCCTCAAAACCCTTGATCAAAACTTTTCAAACCGGCCCTCCAATGCTGGTGCAACCCATTTGGCT *w1-s2*

337 TATGATGCACGGgtaggaatgcagcaccttcatatttttttttattttaaacacaccattaatgtcacttattatatataactatactttctttttgttt IT182932

337 TATGATGCACGGgtaggaatgcagcaccttcatatttttttttattttaaacacaccattaatgtcacttattatatataactatactttctttttgttt *w1-s1*

337 TATGATGCACGGgtaggaatgcagcaccttcatatttttttt-attttaaacacaccattaatgtcacttattatatataactatactttctttttgttt *w1-s2*

437 ttctctctcactaagtgctaaatagaattaaattaacttatgaagaggttagattcggagaattcttataaattaactttacataaattaattttaattt IT182932

437 ttctctctcactaagtgctaaatagaattaaattaacttatgaagaggttagattcggagaattcttataaattaactttacataaattaatttt----- *w1-s1*

436 ttctctctcactaagtgctaaatagaattaaattaacttatgaagaggttagattcggagaattcttataaattaactttacataaattaatttt----- *w1-s2*

537 ataggagaaatttatttattttcttattttttctcctataagtatttattataattttattcaaattagcgcgtgtaaaaaaaaataaatgaactgatcc IT182932

531 ---------------------------------------------------------------------------------------------------- *w1-s1*

530 ---------------------------------------------------------------------------------------------------- *w1-s2*

637 aaaattgaacaaaactctgcttcgaaaaccgaacctttttataagaacggttaggttttagagtggttcaacttgtttctattcaattatttaattagaa IT182932

531 --------------------------------------------------------------------------gtttctattcaattatttaattagaa *w1-s1*

530 --------------------------------------------------------------------------gtttctattcaattatttaattagaa *w1-s2*

737 aaacctatccatatacagtatagaatgaataattgacactaatgcaaccaaaccgaagctaataaaatgtaaaccattttgaaaaaaatttaactaactc IT182932

558 aaacctatccatatacagtatagaatgaataattgacactaatggaaccaaaccgaagctaataaaatgtaaaccattttgaaaaaaatttaactaactc *w1-s1*

557 aaacctatccatatacagtatagaatgaataattgacactaatggaaccaaaccgaagctaataaaatgtaaaccattttgaaaaaaatttaactaactc *w1-s2*

837 actttataaaaa---aaaaaaattgattaaccaaactgtttttatgaggtgatttgtaaatggaccggtttggatttaaatacaaaccaaatgtttcagg IT182932

658 actttataaaaataaaaaaaaattgattaaccaaactgtttttatgaggtgatttgtaaatggaccggtttggatttaaatacaaaccaaatgtttcagg *w1-s1*

657 actttataaaaa---aaaaaaattgattaaccaaactgtttttatgaggtgatttgtaaatggaccggtttggagttaaatacaaaccaaatgtttcagg *w1-s2*

934 gtcttaatccaaaaatgagattcttaaaataagtgaattcttctcttcctctatctatatgtaataatttttctaatatattaaaatacgtagctttata IT182932

758 gtcttaatccaaaaatgagattcttaaaataagtgaattcttctcttcctctatctatatgtaataatttttctaatatattaaaatacgtagctttata *w1-s1*

754 gtcttaatccaaaaatgagattcttaaaataagtgaattcttctcttcctctatctatatgtaataatttttctaatatattaaaatacgtagctttata *w1-s2*

1034 atttactaagataataacatatgtatatcttttcacttttggctattttggatccgtccttgttgacagGATATGGTGTTTGCTCATTACGGATCACGGT IT182932

858 atttactaagataataacatatgtatatcttttcacttttggctattttggatccgtccttgttgacagGATATGGTGTTTGCTCATTACGGATCACGGT *w1-s1*

854 atttactaagataataacatatgtatatcttttcacttttggctattttggatccgtccttgttgacagGATATGGTGTTTGCTCATTACGGATCACGGT *w1-s2*

1134 GGAAGTTGCTAAGAAAACTAAGTAACTTGCACATGCTTGGAGGAAAGGCACTTGATGATTGGGCCCAAATTCGAGATGAAGAGATGGGGCACATGCTTGG IT182932

958 GGAAGTTGCTAAGAAAACTAAGTAACTTGCACATGCTTGGAGGAAAGGCACTTGATGATTGGGCCCAAATTCGAGATGAAGAGATGGGGCACATGCTTGG *w1-s1*

954 GGAAGTTGCTAAGAAAACTAAGTAACTTGCACATGCTTGGAGGAAAGGCACTTGATGATTGGGCCCAAATTCGAGATGAAGAGATGGGGCACATGCTTGG *w1-s2*

1234 TGCAATGTACGATTGTAACAAGAGGGATGAGGCTGTGGTGGTGGCGGAGATGTTGACATATTCAATGGCCAACATGATTGGCCAAGTTATATTGAGTCGT IT182932

1058 TGCAATGTACGATTGTAACAAGAGGGATGAGGCTGTGGTGGTGGCGGAGATGTTGACATATTCAATGGCCAACATGATTGGCCAAGTTATATTGAGTCGT *w1-s1*

1054 TGCAATGTACGATTGTAACAAGAGGGATGAGGCTGTGGTGGTGGCGGAGATGTTGACATATTCAATGGCCAACATGATTGGCCAAGTTATATTGAGTCGT *w1-s2*

1334 CGAGTGTTTGAGACAAAGGGTTCGGAGTCTAACGAGTTCAAGGACATGGTGGTTGAGCTCATGACCGTTGCTGGTTACTTCAACATTGGTGACTTCATAC IT182932

1158 CGAGTGTTTGAGACAAAGGGTTCGGAGTCTAACGAGTTCAAGGACATGGTGGTTGAGCTCATGACCGTTGCTGGTTACTTCAACATTGGTGACTTCATAC *w1-s1*

1154 CGAGTGTTTGAGACAAAGGGTTCGGAGTCTAACGAGTTCAAGGACATGGTGGTTGAGCTCATGACCGTTGCTGGTTACTTCAACATTGGTGACTTCATAC *w1-s2*

1434 CCTTTTTGGCCAAGTTGGACTTGCAAGGCATAGAGCGTGGCATGAAGAAGTTGCACAAGAAGTTTGATGCGTTGTTAACGAGCATGATTGAGGAGCATGT IT182932

1258 CCTTTTTGGCCAAGTTGGACTTGCAAGGCATAGAGCGTGGCATGAAGAAGTTGCACAAGAAGTTTGATGCGTTGTTAACGAGCATGATTGAGGAGCATGT *w1-s1*

1254 CCTTTTTGGCCAAGTTGGACTTGCAAGGCATAGAGCGTGGCATGAAGAAGTTGCACAAGAAGTTTGATGCGTTGTTAACGAGCATGATTGAGGAGCATGT *w1-s2*

1534 TGCTTCTAGTCACAAGAGAAAGGGCAAGCCCGATTTCTTAGACATGGTAATGGCTCATCATAGTGAGAACTCCGATGGGGAGGAACTATCGCTCACCAAC IT182932

1358 TGCTTCTAGTCACAAGAGAAAGGGCAAGCCCGATTTCTTAGACATGGTAATGGCTCATCATAGTGAGAACTCCGATGGGGAGGAACTATCGCTCACCAAC *w1-s1*

1354 TGCTTCTAGTCACAAGAGAAAGGGCAAGCCCGATTTCTTAGACACGGTAATGGCTCATCATAGTGAGAACTCCGATGGGGAGGAACTATCGCTCACCAAC *w1-s2*

1634 ATCAAGGCACTACTCTTGgtataacgctttttatcttacttctcaaatgtgtcattttctttcttcatttttattagacaaaaaaaaaaagtaaaatatt IT182932

1458 ATCAAGGCACTACTCTTGgtataacgctttttatcttacttctcaaatgtgtcattttctttcttcatttttattagacaaaaaaaaaa-gtaaaatatt *w1-s1*

1454 ATCAAGGCACTACTCTTGGTATAAcgctttttatcttacttctcaaatgtgtcattttctttcttcatttttattagacaaaaaaaaaaagtaaaatatt *w1-s2*

1734 tgttatatgaggataactaccatggaggatcacttatggtatccaacgttgttaaataaccgttataatccgccttaacgttataacgtggcatttttca IT182932

1557 tgttatatgaggataactaccatggaggatcacttatggtatccaacgttgttaaataaccgttataatccgccttaacgttataacgtggcatttttca *w1-s1*

1554 tgttatatgaggataactaccatggaggatcacttatggtatccaacgttgttaaataaccgttataatccgccttaacgttataacgtggcatttttca *w1-s2*

1834 acccctctgcccatagacagtttgtgagggaggcccgttatgactgcgccatagggtgcaatggcttcctaata-ggagaaattttagccttctgcatat IT182932

1657 acccctctgcccatagacagtttgtgagggaggcccgttatgacggcgccatagggtgcaatggcttcctaatatggagaaattttagccttctgcatat *w1-s1*

1654 acccctctgcccatagacagtttgtgagggaggcccgttatgactgcgccatagggtgcaatggcttcctaata-ggagaaattttagccttctgcatat *w1-s2*

1933 gtcgtcatctgtcattgataactttggtgtggtgttaacaaaacaacttatatagttaggataggtagtaaaaagaaagtgttatttccatatttttcaa IT182932

1757 gtcgtcatctgtcattgataactttggtgtggtgttaacaaaacaacttatatagttaggataggtagtaaaaagaaagtgttatttccatatttttcaa *w1-s1*

1753 gtcgtcatctgtcattgataactttggtgtggtgttaacaaaacaacttatatagttaggataggtagtaaaaagaaagtgttatttccatatttttcaa *w1-s2*

2033 accccttgttatatatatatag--tggcagacctacatgagtgataaaaaaatttattctcttcataaaaaagtgtaggagggtacttgtacctccttat IT182932

1857 accccttgttatatatatatag--tggcggacctacatgagtgataaaaaaatttattctcttcataaaaaagtgtaggagggtacttgtacctccttat *w1-s1*

1853 atcccttgttatatatatatatattggcggacctacatgagtgataaaaaaatttattctcttcataaaaaagtgtaggagggtacttgtacctccttat *w1-s2*

2131 tttttaaaatttattaaatttataaataaaattttatatttttatattttattttatctatatttatataattaaatccactaattttattttttataat IT182932

1955 tttttaaaatttattaaatttataaataaaattttatatttttatattttattttatctatatttatataattgaatccactaattttattttttataat *w1-s1*

1953 tttttaaaatttattaaatttataaataaaattttatatttttatattttattttatctatatttatataattgaatccactaattttattttttataat *w1-s2*

2231 ttaaactccctaatttagagtcttggatccgcc------------------------------------------------------------------- IT182932

2055 ttaaactccctaatttaaa-tcttggatccgcc------------------------------------------------------------------- *w1-s1*

2053 ttaaactcactaatttagagtcttggatccgccatatatatatatatatatatatatatatatatatatatatatatatatatatatatatatatatata *w1-s2*

2263 ------------------tatatatatatatatcaatcgtttttttacattttcaagaattatattaaaaacttcaacatctttaattcaaaaatgctat IT182932

2086 --------------------tatatatatatatcaatggtttttttacattttcaagaattatattaaaaacttcaacatcttttattcaaaaatgctat *w1-s1*

2153 tatatatatatatatatatatatatatatatatcaatggtttttttacattttcaagaattatattaaaaacttcaacatctttaattcaaaaatgctat *w1-s2*

2346 acactttctaattcattcttttaaacactttctaattcattttttttaaatatattattattgactaaaattgattgtaaatcatacattggttctattt IT182932

2167 acactttctaattcattcttttaaacactttctaattcattttttt-aaatatattattattgactaaaattgattgtaaatcatacattggttctattt *w1-s1*

2253 acactttctaattcattcttttaaacactttctaattcattttttt-aaatatattattattgactaaaattgattgtaaatcatacattggttctattt *w1-s2*

2446 tttttattgaatgagtctcacttgttctgtggtttctaacatattttaactaatatcaaagagtaggtacgttgagtgtattgttggttcaattcttttc IT182932

2266 tttttattgaatgagtctcacttgttctgtggtttctaacatattttaactaatatcaaagagtaggtacgttgagtgtattgttggttcaattcttttc *w1-s1*

2352 tttttattgaatgagtctcacttgttctgtggtttctaacatattttaactaatatcaaagagtaggtacgttgagtgtattgttggttcaattcttttc *w1-s2*

2546 tttttataaaaatttttgatatcataatatttgaacgttttgtttaaaataatcttccctctatttgtacataattatagcatgtttgttttggcaaatt IT182932

2366 tttttataaaaaattttgatatcataatatttgaaagttttgtttaaaataatcttccctctatttgtacataattatagcatgtttgttttggcaaatt *w1-s1*

2452 tttttataaaattttttgatatcataatatttgaaagttttgtttaaaataatcttccctctatttgtacataattatagcatgtttgttttggcaaatt *w1-s2*

2646 gaataaaaagtgaaaaatttggaagcaatataagacttcgttagaaccattaaaaaaacataatcaatttcccttcgccacccccacacacatacatagt IT182932

2466 gaataaaaagtgaaaaatttggaagcaatataaaacttcgttagaaccattaaaaaaacataatcaatttcccttcgccacccccacacacatacatagt *w1-s1*

2552 gaataaaaagtgaaaaatttggaagcaatataagacttcgttagaaccattaaaaaaacataatcaatttcccttcgccacccccacacacatacatagt *w1-s2*

2746 aaatttagtcctacacatcataacttattttgcctgaaaaatgttgagttaatttttatgacttgaagtgacaaaaatacgttcaaaatttgtttatatt IT182932

2566 aaatttagtcctacacatcataacttattttgcctgaaaaatgttgagttaatttttatgacttgaagtgacaaaaatacgttcaaaatttgtttatatt *w1-s1*

2652 aaatttagtcctacacatcataacttattttgcctgaaaaatgttgagttaatttttatgacttgaagtgacaaaaatacgttcaaaatttgtttatatt *w1-s2*

2846 gttcaaactataaatttacaattgaaccacaaagaaaaaggatttccgtatgacaaattaaaaattaattgcgatattgcatagttaactctactatatc IT182932

2666 gttcaaactataaatttacaattgaaccacaaagaaaaaggatttccgtatgacaaattaaaaattaattgcgatattgcatagttaactctactatatc *w1-s1*

2752 gttcaaactataaatttacaattgaaccacaaagaaaaaggatttccgtatgacaaattaaaaattaattgcgatattgcatagttaactctactatatc *w1-s2*

2946 tgaatttttatttgttttacaagtacaacttgtttatgatataagtttagctataagccaagtaagtacactttgaatttagccaaaaaggaaatgggca IT182932

2766 tgaatttttatttgttttacaagtacaacttgtttatgatataagtttagctataagccaagtaagtacactttgaatttagccaaaaaggaaatgggca *w1-s1*

2852 tgaatttttatttgttttacaagtacaacttgtttatgatataagtttagctataagccaagtaagtacactttgaatttagccaaaaaggaaatgggca *w1-s2*

3046 gtctgtatcataaattttcttagacggaaatattaaagtacaagctacgaatatatcgtatatattgtgtgagatcaacttaaattaatcatgatggagg IT182932

2866 gtctgtatcataaattttcttagacggaaatattaaagtacaagctacgaatatatcgtatatattgtgtgagatcaacttaaattaatcatgatggagg *w1-s1*

2952 gtctgtatcataaattttcttagacggaaatattaaaatacaagctacgaatatatcgtatatattgtgtgagatcaacttaaattaatcatgatggagg *w1-s2*

3146 ttaaatgctgcaattaaattaaattcagcgggcctctcccccaattatttataccaactttttgctgcatttggaattggggccacggaaagtaatgtcc IT182932

2966 ttaaatgctgcaattaaattaaattcagcgggcctctcccccaattatttataccaactttttgctgcatttggaattggggccacggaaagtaatgtcc *w1-s1*

3052 ttaaatgctgcaattaaattaaattcagcgggcctctcccccaattatttataccaactttttgctgcatttggaattggggccacggaaagtaatgtcc *w1-s2*

3246 caactaagaaaatatcttctcatcatttggtattgtacgtagtgaatcacattgactatatatcatgtattaaatctgatatgagaatatttattttcca IT182932

3066 caactaagaaaatatcttctcatcatttggtattgtacgtagtgaatcacattgactatatatcatgtattaaatctgatatgagaatatttattttcca *w1-s1*

3152 caactaagaaaatatcttctcatcatttggtattgtacgtagtgaatcacattgactatatatcatgtattaaatctgatatgagaatatttattttcca *w1-s2*

3346 tcttattttctatatgcataataatattagtttttgtctagtatatatatcacatttttaatacataaataacaaatttagtcaagacttttttttttaa IT182932

3166 tcttattttctatatgcataataatattagtttttgtctagtatatatatcacatttttaatacataaataacaaatttagtcaacacttttttttttaa *w1-s1*

3252 tcttattttctatatgcataataatattagtttttgtctagtatatatatcacatttttaatacataaataacaaatttagtcaagacttttttttttaa *w1-s2*

3446 aaaaaaaagacctaaaattttgtttacactagaaactaaatattaattgttgtgactaaattacaatgtgaatataataataccatcataatagtgttca IT182932

3266 aaaaaaaagacctaaaattttgtttacactagaaactaaatattaattgttgtgactaaattacaatgtgaatataataataccatcataatagtgttca *w1-s1*

3352 aaaaaaaagacctaaaattttgtttacactagaaactaaatattaattgttgtgactaaattacaatgtgaatataataataccatcttaatagtgttca *w1-s2*

3546 attttaacaaaaaaatct--gttatatatagtgcaaattcaacgaatcaatacaaatcatattttatataaaaatttattgatgatgtaaatgttagtgc IT182932

3366 attttaacaaaaaaatctatgttatatatagtgcaaattcaacgaatcaatacaaatcatattttatataaaaatttattgatgatgtaaatgttagtgc *w1-s1*

3452 attttaacaaaaaaatctatgttatatatagtgcaaattcaacgaatcaatacaaatcatattttatataaaaatttattgatgatgtaaatgttagtgc *w1-s2*

3644 aagttattacgatgataatttaatccctcgcctcataatcataccacacaccaacattttctagcttgagattttgttctaacaactatatatgctattt IT182932

3466 aagttattacgatgataatttaatccctcgcctcataatcataccacacaccaacattttctagcttgagattttgttctaacaactatatatgctattt *w1-s1*

3552 aagttattacgatgataatttaatccctcgcctcataatcataccacacaccaacattttctagcttgagattttgttctaacaactatatatgctattt *w1-s2*

3744 tgttccagAACCTATTCACCGCAGGCACCGATACATCTTCAAGTATAATAGAGTGGTCCTTAGCCGAGATGTTGAAGAAGCCCAGCATAATGAAGAAGGC IT182932

3566 tgttccagAAACTATTCACCGCAGGCACCGATACATCTTCAAGTATAATAGAGTGGTCCTTAGCCGAGATGTTGAAGAAGCCCAGCATAATGAAGAAGGC *w1-s1*

3652 tgttccagaacctattcaccgcaggcaccgatacatcttcaagtataatagagtggtccttagccgagatgttgaagaagcccagcataatgaagaaggc *w1-s2*

3844 TCATGAAGAAATGGACCAAGTCATAGGAAGGGATCGCCGTCTCAAAGAATCTGACATACCAAAGCTTCCCTACTTCCAAGCCATTTGCAAAGAGACCTAT IT182932

3666 TCATGAAGAAATGGACCAAGTCATAGGAAGGGATCGCCGTCTCAAAGAATCTGACATACCAAAGCTTCCCTACTTCCAAGCCATTTGCAAAGAGACCTAT *w1-s1*

3752 tcatgaagaaatggaccaagtcataggaagggatcgccgtctcaaagaatctgacataccaaagcttccctacttccaagccatttgcaaagagacctat *w1-s2*

3944 AGAAAGCACCCTTCAACACCCCTAAACCTGCCTCGAATCTCATCTGAACCGTGCCAAGTGAATGGTTACTACATTCCCGAGAACACTAGGCTGAATGTGA IT182932

3766 AGAAAGCACCCTTCAACACCCCTAAACCTGCCTCGAATCTCATCTGAACCGTGCCAAGTGAATGGTTACTACATTCCCGAGAACACTAGGCTGAATGTGA *w1-s1*

3852 agaaagcacccttcaacacccctaaacctgcctcgaatctcatctgaaccgtgccaagtgaatggttactacattcccgagaacactaggctgaatgtga *w1-s2*

4044 ACATTTGGGCCATAGGAAGAGACCCTGATGTGTGGAACAATCCTTTGGAGTTTATGCCCGAGAGGTTTTTGAGTGGGAAGAATGCCAAAATTGACCCACG IT182932

3866 ACATTTGGGCCATAGGAAGAGACCCTGATGTGTGGAACAATCCTTTGGAGTTTATGCCCGAGAGGTTTTTGAGTGGGAAGAATGCCAAAATTGACCCACG *w1-s1*

3952 acatttgggccataggaagagaccctgatgtgtggaacaatcctttggagtttatgcccgagaggtttttgagtgggaagaatgccaaaattgacccacg *w1-s2*

4144 TGGGAATGATTTTGAGCTTATTCCATTTGGTGCTGGGAGGAGGATTTGTGCAGGGACTAGGATGGGGATTGTGTTGGTTCACTACATTTTGGGCACTTTG IT182932

3966 TGGGAATGATTTTGAGCTTATTCCATTTGGTGCTGGGAGGAGGATTTGTGCAGGGACTAGGATGGGGATTGTGTTGGTTCACTACATTTTGGGCACTTTG *w1-s1*

4052 tgggaatgattttgagcttattccatttggtgctgggaggaggatttgtgcagggactaggatggggattgtgttggttcactacattttgggcactttg *w1-s2*

4244 GTGCATTCGTTTGATTGGAAGCTACCCAATGGGGTGAGGGAGTTAGACATGGAGGAGTCCTTTGGGCTTGCCTTGCAAAAAAAGGTTCCACTTGCTGCTT IT182932

4066 GTGCATTCGTTTGATTGGAAGCTACCCAATGGGGTGAGGGAGTTAGACATGGAGGAGTCCTTTGGGCTTGCCTTGCAAAAAAAGGTTCCACTTGCTGCTT *w1-s1*

4152 gtgcattcgtttgattggaagctacccaatggggtgagggagttagacatggaggagtcctttgggcttgccttgcaaaaaaaggttccacttgctgctt *w1-s2*

4344 TGGTTACCCCTAGGTTGAACCCAAGTGCTTACATTTCTTAGaattggttgggttcgaatattcaccagctatgttctctagccttattttgttgtccaat IT182932

4166 TGGTTACCCCTAGGTTGAACCCAAGTGCTTACATTTCTTAGaattggttgggttcgaatattcaccagctatgttctctagccttattttgttgtccaat *w1-s1*

4252 tggttacccctaggttgaacccaagtgcttacatttcttagaattggttgggttcgaatattcaccagctatgttctctagccttattttgttgtccaat *w1-s2*

4444 gattttgtggctgtggctacataaataagtaatgtttgggttgcacaacctatttgtatttgtaaggttctatgttacttggaaatccgtt IT182932

4266 gattttgtggctgtggctacataaataagtaatgtttgggttgcacaacctatttgtatttgtaaggttctatgttacttggaaatccgtt *w1-s1*

4352 gattttgtggctgtggctacataaataagtaatgtttgggttgcacaacctatttgtatttgtaaggttctatgttacttggaaatccgtt *w1-s2*

**S3 Fig.** **Alignment of *F3′5′H* genomic sequences from IT182932, *w1-s1*, and *w1-s2*.**
