# Supplementary figures and images for: Identification and Molecular Analysis of Four New Alleles at the W1 Locus Associated with Flower Color in Soybean
Source: PLoS One. 2016 Jul 21;11(7):e0159865. doi: 10.1371/journal.pone.0159865 (PMC4956318; doi:10.1371/journal.pone.0159865)

A

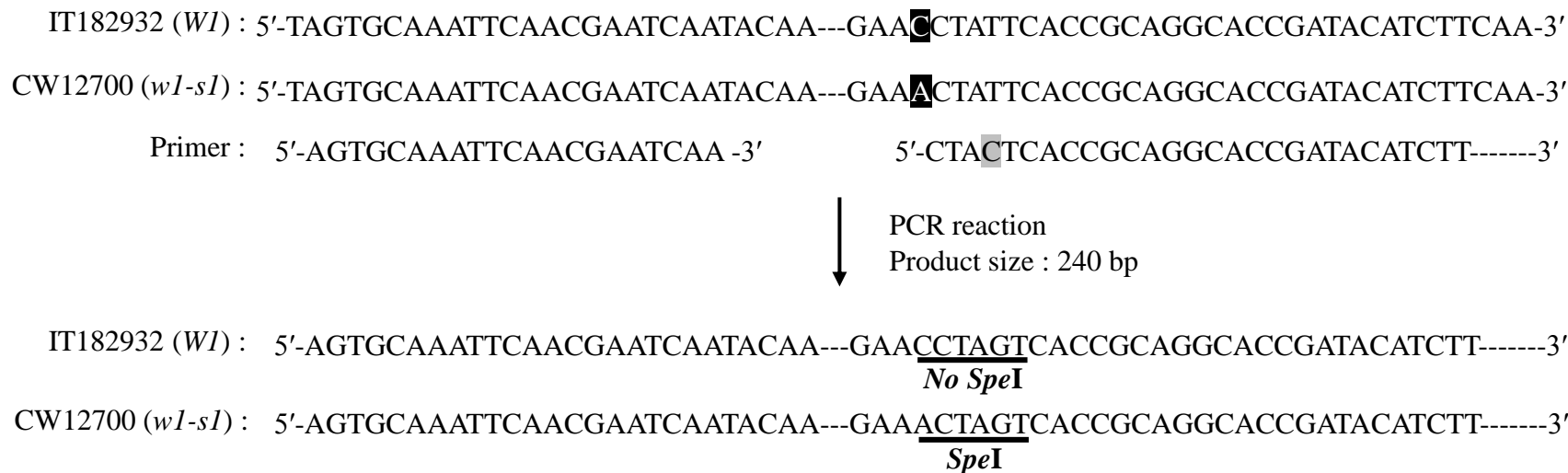

B

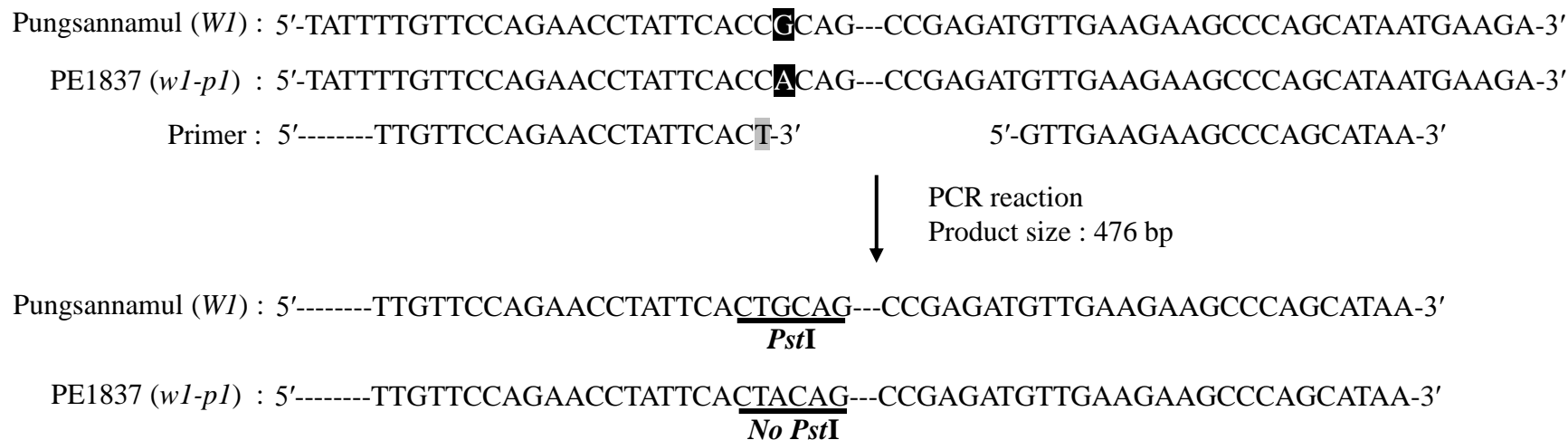

C

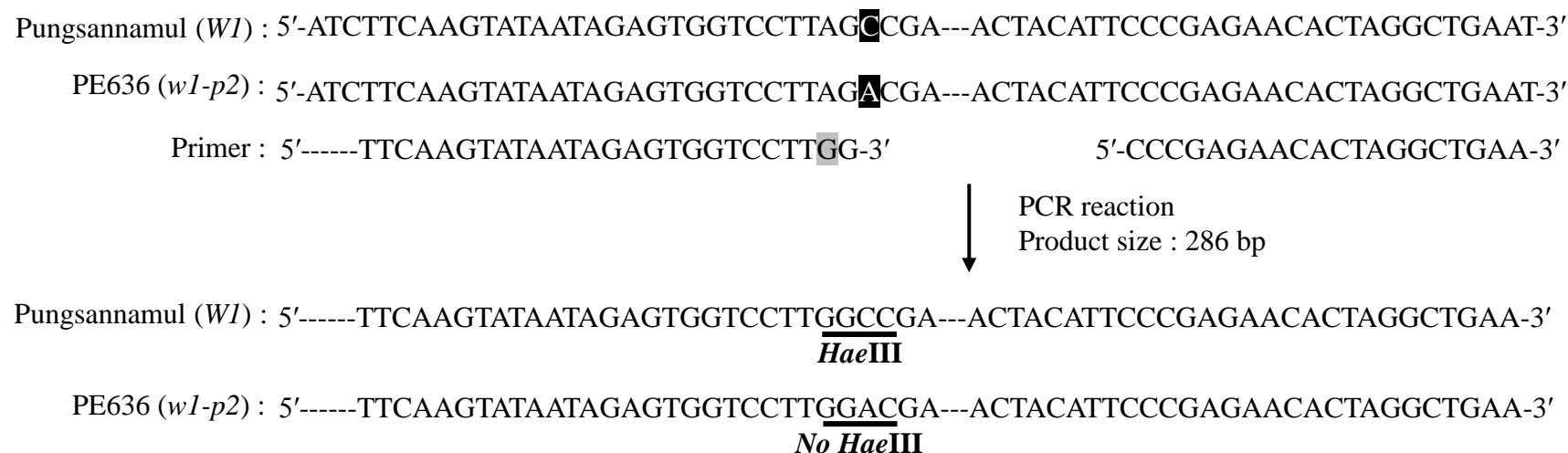

**S4 Fig. Schematic diagrams of dCAPS analysis to detect a SNPs in *w1-s1*, *w1-p1* and *w1-p2*.**

Supplement: S4 Fig — (A) Diagram of dCAPS analysis to detect a SNP in the w1-s1 allele (highlighted in black). The SpeI site (ACTAGT) underlined was artificially introduced in the PCR products of the w1-s1 allele by using a reverse primer with a mismatched base (C, highlighted in grey). The PCR products from IT182932 are 240-bp in length and not digested with SpeI, whereas those from w1-s1 are 194-bp in length after digestion. (B) Diagram of dCAPS analysis to detect a SNP in the w1-p1 allele (highlighted in black). The PstI site (CTGCAG) underlined was artificially introduced in the PCR products of the W1 allele by using a forward primer with a mismatched base (T, highlighted in grey). The PCR products from PE1837 (w1-p1) are 476-bp in length and not digested with PstI, whereas those from W1 are 431-bp in length after digestion. (C) Diagram of dCAPS analysis to detect a SNP in the w1-p2 allele (highlighted in black). The HaeIII site (GGCC) underlined was artificially introduced in the PCR products of the W1 allele by using a forward primer with a mismatched base (G, highlighted in grey). The PCR products from PE636 (w1-p2) are 286-bp in length and not digested with HaeIII, whereas those from W1 are 231-bp in length after digestion. (PDF) [file pone.0159865.s004.pdf]
